# Inhibition of focal adhesion kinase enhances antitumor response of radiation therapy in pancreatic cancer through CD8+ T cells

**DOI:** 10.20892/j.issn.2095-3941.2020.0273

**Published:** 2021-02-15

**Authors:** Arsen Osipov, Alex B. Blair, Juliane Liberto, Jianxin Wang, Keyu Li, Brian Herbst, Yao Xu, Shiqi Li, Nan Niu, Rufiaat Rashid, Ding Ding, Yanan Liu, Zaiqi Wang, Christopher L. Wolfgang, Richard A. Burkhart, Daniel Laheru, Lei Zheng

**Affiliations:** 1 The Sydney Kimmel Comprehensive Cancer Center, Johns Hopkins University School of Medicine, Baltimore 21287, MD, USA; 2 The Pancreatic Cancer Precision Medicine Center of Excellence Program, Johns Hopkins University School of Medicine, Baltimore 21287, MD, USA; 3 Department of Oncology, Johns Hopkins University School of Medicine, Baltimore 21287, MD, USA; 4 Department of Surgery, Johns Hopkins University School of Medicine, Baltimore 21287, MD, USA; 5 InxMed Shanghai, Shanghai 201202, China

**Keywords:** Focal adhesion protein-tyrosine kinases, radiotherapy, pancreatic neoplasms, immunomodulation

## Abstract

**Objective:**

Pancreatic ductal adenocarcinoma (PDAC) is a deadly malignancy, due in large part to its resistance to conventional therapies, including radiotherapy (RT). Despite RT exerting a modest antitumor response, it has also been shown to promote an immunosuppressive tumor microenvironment. Previous studies demonstrated that focal adhesion kinase inhibitors (FAKi) in clinical development inhibit the infiltration of suppressive myeloid cells and T regulatory (T regs) cells, and subsequently enhance effector T cell infiltration. FAK inhibitors in clinical development have not been investigated in combination with RT in preclinical murine models or clinical studies. Thus, we investigated the impact of FAK inhibition on RT, its potential as an RT sensitizer and immunomodulator in a murine model of PDAC.

**Methods:**

We used a syngeneic orthotopic murine model to study the effect of FAKi on hypofractionated RT.

**Results:**

In this study we showed that IN10018, a small molecular FAKi, enhanced antitumor response to RT. Antitumor activity of the combination of FAKi and RT is T cell dependent. FAKi in combination with RT enhanced CD8+ T cell infiltration significantly in comparison to the radiation or FAKi treatment alone (*P* < 0.05). FAKi in combination with radiation inhibited the infiltration of granulocytes but enhanced the infiltration of macrophages and T regs in comparison with the radiation or FAKi treatment alone (*P* < 0.01).

**Conclusions:**

These results support the clinical development of FAKi as a radiosensitizer for PDAC and combining FAKi with RT to prime the tumor microenvironment of PDAC for immunotherapy.

## Introduction

Among all malignancies, pancreatic ductal adenocarcinoma (PDAC) is one of the most treatment resistant and deadliest. It is estimated that by the year of 2030, PDAC will become the second leading cause of cancer related death in the United States^1^. Currently, the treatment for pancreatic cancer typically involves three modalities: surgery, cytotoxic chemotherapy, and radiation therapy (RT). However, the role of RT is not fully established in PDAC due to its modest effects^2,3^. Additionally, clinical trials evaluating the addition of RT to surgery and chemotherapy have had mixed results^4–9^. This is in large part due to the resistance of PDAC to conventional therapies, including RT^10,11^.

How to sensitize PDAC to radiotherapy has been an active area of research. Beyond the canonical effect of radiation-induced cell death on cancer, antitumor effects mediated by RT have been shown to be T cell dependent^12^. Currently, radiosensitization in PDAC is achieved by employing cytotoxic chemotherapy^13^. However, the use of conventional cytotoxic chemotherapy as a means of radiosensitization leads to increased off-tumor cell toxicity as evidenced by CD8+ T cell depletion^14,15^. Consistently, our group previously found that a poorer outcome of PDAC patients following RT, particularly, stereotactic body radiation (SBRT), is associated with suppressed systemic lymphocyte counts^14^. Although the literature suggested RT augments anti-tumor immunity, published studies also showed that it can promote the immunosuppressive tumor microenvironment (TME) including tumor PD-L1 expression, increased immunosuppressive myeloid phenotypes [myeloid derived suppressor cells (MDSC), tumor associated neutrophils] and T regulatory cells (T reg), which are anticipated to dampen CD8+ T cell response and clinical efficacy^16–19^.

Focal adhesion kinase (FAK), a non-receptor tyrosine kinase, mediates a multitude of cellular and extracellular processes involved in tumor cell adhesion, invasion, and metastases^20^. It is also implicated as a potential molecular target for TME immunomodulation. Jiang and colleagues^21^ have recently shown that inhibition of FAK sensitizes tumors to chemotherapy and immune checkpoint inhibitor by reducing immunosuppressive cells [MDSCs, tumor associated macrophages (TAM), T reg] and consequently impacting effector T cell infiltration into the TME of PDAC KPC mouse models. It was also shown that FAK inhibition sensitizes tumors for RT in the preclinical models of other malignancies including non-small cell lung cancer (NSCLC), head and neck squamous cell carcinoma (HNSCC), and breast cancer^22–24^. Previously, *in vitro* work has suggested that FAK knockdown can impact radiation survival and pancreatic cell proliferation^25^. However, whether FAK inhibition can sensitize PDAC to RT has not been investigated in a preclinical murine model.

Currently, FAK inhibitors are under clinical development across numerous tumor types, including PDAC^20^. In metastatic PDAC, the active phase I study of combination therapy with chemotherapy, a small molecule FAK inhibitor defactinib, and an anti-PD-1 antibody pembrolizumab, has been shown to be safe and to not significantly increase toxicity relative to cytotoxic chemotherapy alone (NCT02546531)^26^. Preliminary data from this study has also indicated that treatment with combination therapy with FAKi, is associated with decreased p-FAK expression and increased CD8+ T cell infiltration in PDAC^26^. A novel, potent, and highly selective FAKi, BI-853520, also known as IN10018, has recently been shown to exert anti-tumor effects in multiple cancer types including breast, esophageal, head and neck, gastric, lung, ovarian, pancreatic, and prostate cancers *in vivo*^27–29^. The efficacy of IN10018 in adenocarcinoma xenograft models is linked to a mesenchymal tumor phenotype^28^. It has also recently been evaluated in a phase I study, which included 17 patients with PDAC, who had manageable and acceptable toxicity profiles with some achieving durable stable disease^30^. However, these FAK inhibitors in clinical development have not been investigated in combination with RT in preclinical or clinical studies, and how these FAK inhibitors in combination with RT may modulate the TME remain unknown.

Therefore, in this current investigation we sought to understand the impact of FAK inhibition by using IN10018 on radiotherapy treatment and investigate its potential as a radiotherapy sensitizer and immune modulator in a murine orthotopic model of PDAC.

## Materials and methods

### Cell lines

KPC tumor cells (KrasLSL.G12D/+; p53R172H/+; PdxCretg/+) are an established PDAC cell line derived from a C57Bl/6 background mouse model. This model utilizes a cre recombinase under the control of the pancreas-specific pdx-1 promoter and floxed KRASG12D in combination with mutated p53 (Trp53R172H/+) expression. The cell lines were developed and cultured as previously described and were validated by short-tandem-repeat profiling and testing for mycoplasma every 6 months^31^. KPC cells were maintained at 37 °C in 5% carbon dioxide (CO_2_) with RMPI 1640 media (Life Technologies, Carlsbad, CA, USA) supplemented with 10% heat-inactivated fetal bovine serum (HI-FBS; Benchmark, Sacramento, CA, USA), 1% penicillin/streptomycin (pen/strep; Life Technologies), 1% MEM Non-Essential Amino Acids Solution (MEM-NEAA; Life Technologies), 1% sodium pyruvate (Sigma, St. Louis, MO, USA) and 1% L-glutamine (Life Technologies).

### Mice and *in vivo* experiments

Syngeneic C57Bl/6 female mice (6–8 weeks) were purchased from Harlan Laboratories (Frederick, MD, USA) and maintained in accordance with the Institutional Animal Care and Use Committee (IACUC) of Johns Hopkins University and maintained according to the American Association of Laboratory Animal Care guidelines and in line with our animal protocol (MO19M133). Mice considered to have reached a “survival endpoint” including hunched posture, lethargy, dehydration, and rough hair coat were euthanized. The IACUC mouse protocol was maintained by third-party management.

Procedures for the orthotopic model were modified from our previous report^32^. PDAC cells (2 × 10^6^) of the KPC cell line were injected subcutaneously into the flanks of syngeneic female C57Bl/6 mice. After 1 to 2 weeks, the subcutaneous tumors were harvested and cut into approximately 2 mm^3^ pieces. The orthotopic tumor inoculation, with surgical clips placed around the tumor to guide the RT, was performed on female C57Bl/6 mice at ages 6–8 weeks, on day 0 as described previously^33^.

FAK inhibitor, IN10018, supplied by InxMed, was administered by oral gavage 50 mg/kg, once daily beginning on day 6 following surgery. For RT, mice were anesthetized with isoflurane and pancreas tumors were irradiated with 8 Gy × 3 fractions delivered daily between day 13 and day 15 after the surgery, at a dose rate of 3 Gy/min using the Small Animal Radiation Research Platform (SARRP; Xstrahl, Suwanee, GA, USA). The choice of the RT dosing/schedule (8 Gy × 3 days) resemble the SBRT 6.5 Gy × 5 days commonly used for human PDAC patients^34^ and is also considered to be the most optimal immunogenic dose in the literature^35^. We have tested this dose/schedule in our previous study^33^. The isocenter was placed at the center of the fiducials for RT. Tumor size was monitored weekly using small-animal ultrasound (US) (Vevo770; VisualSonics, Toronto, Canada). Visualization of both sagittal and transverse planes were used to calculate tumor size and volume. CD8+ T cell depletion experiments were conducted with anti-CD8a (clone 2.43; BioXCell, Lebanon, New Hampshire, USA) given 10 mg/kg per mouse was delivered twice weekly beginning day 3 following surgery until day 30.

### Cell processing

Dissected orthotopic pancreatic tumors were collected following mice sacrifice on day 20 from tumor inoculation for analysis of tumor infiltrating lymphocytes (TILs). Each tumor was digested by using a tumor dissociation kit and processed sequentially through 40-µm and 100-µm nylon filters and brought to a volume of 20 mL of CTL medium. Suspensions were centrifuged at 1,500 rpm for 5 min. Cell pellets were suspended in 4 mL of ammonium-chloride-potassium (ACK) lysis (Quality Biological) and subsequently spun at 1,500 rpm for 5 min. Cell pellets were then resuspended in 6 mL 80% Percoll (GE Healthcare LifeSciences), overlaid with 6 mL 40% Percoll and centrifuged at room temperature for 25 min at 3,200 rpm without brake. The lymphocyte layer was removed and quenched with 30 mL of CTL media. Isolated TILs were enriched for CD11b+ cells using the EasySep™ Mouse CD11b Positive Selection Kit (Stemcell Technologies).

### Flow cytometry

Processed cells from each mouse were stained with Live Dead Aqua Dead Cell Kit (Invitrogen) for 30 min on ice, washed with phosphate-buffered saline (PBS) and then blocked with rat anti-mouse Fc antibody (CD16/CD32 clone 2.4G2; BD Biosciences, San Diego, CA, USA) in FACs buffer for 10 min. Following blocking, cells were stained for the following anti-mouse fluorophores for 1 h on ice: CD45-PERCP/Cy5.5, CD3-APC Cy7 (Biolegend, San Diego, CA, USA), CD4-APCFire750 (Biolegend), CD8a AF700 (Biolegend), FoxP3-PE (ebioscience), CD25-BV421 (Biolegend), CD11b-BV650 (Biolegend), Ly6C-PE-Dazzle 594 (Biolegend), Ly6G-FITC (Biolegend), and F4/80-PE/Cy7 (ebioscience, San Diego, CA, USA). Cells were then washed, resuspended in FACs buffer and assayed on a Cytoflex flow cytometer (Beckman Coulter, Pasadena, CA, USA). FACs buffer consisted of HBSS (Sigma) with 2% bovine calf serum (Sigma), 0.1% sodium azide (Sigma), and 0.1% HEPES.

The “total number” of immune cells was determined by the end gated count by FACS analysis of the single cell suspension and represent the absolute number of cells infiltrating in the entire analyzed tissue. The “percentage” of immune cells represents the percentage of an immune cell subtype gated among the gated live immune cell counts as determined by Live Dead Aqua and the plot of side scatter *vs*. forward scatter. Gating for myeloid phenotypes shown in **Supplementary Figure S1**.

### Statistical analysis

Statistical analyses and graphing were performed using GraphPad Prism software (GraphPad Software, San Diego, CA, USA). For comparison of cell number or percentage, one-way analysis of variance (ANOVA) or Student’s *t* tests were applied to compare the mean value among or between groups. Kaplan–Meier curves and log-rank tests were performed for survival estimates and to analyze survival outcomes between subgroups. A two-way ANOVA with Šidák correction was used for multiple comparisons of tumor volume. A *P*-value of < 0.05 was considered statistically significant.

## Results

### FAKi functions as a radiosensitizer enhancing RT tumor reduction in murine PDAC

**Figure 1:**
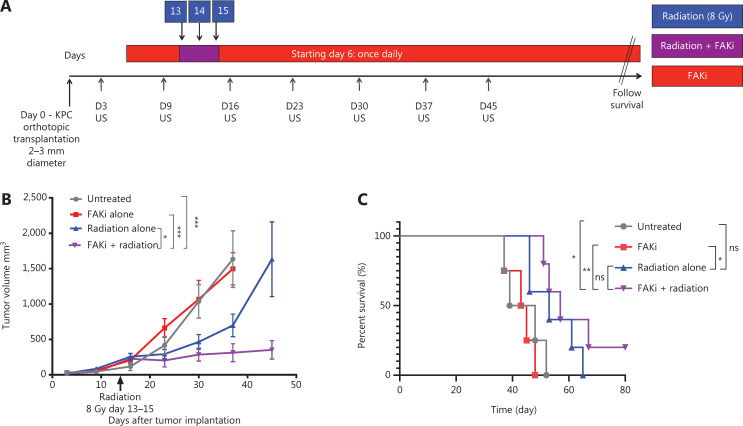
FAKi enhances RT antitumor response and survival in a murine model of PDAC. Pancreatic adenocarcinoma was established *via* KPC tumor cell inoculation by orthotopic implantation on Day 0. Mice were then treated with FAKi (IN10018, 50 mg/kg) by oral gavage once daily beginning on day 6 following surgery. Fractions of hypofractionated radiation (8 Gy × 3) were delivered daily between day 13 and day 15 after the surgery using surgical clips as radiation field markers. US measurements in three dimensions were taken on days 3, 9, 16, 23, 30, 37, and 45. Mice were then followed for survival. (A) Experimental design and treatment schema. (B) Tumor volume, represented as mm^3^, on the y axis was calculated using three dimensional (3D) US measurements within the transverse and sagittal planes. US measurements in three dimensions was taken on days 3, 9, 16, 23, 30, 37, and 45 as noted on the x axis. Each treatment group contained four to five mice. Data points represent mean ± SEM at each US measurement time point from one representative experiment. A two-way ANOVA with Šidák multiple comparisons test for each point was used. (C) Kaplan–Meier survival curves of mice with orthotopic implanted PDAC KPC cells in untreated mice (*n* = 4), mice treated with FAKi alone (*n* = 4), RT alone (*n* = 5), or the combination FAKi and RT treatment (*n* = 5). ns, not significant, *P < 0.05, **P < 0.01, ***P < 0.001, by log-rank test.

To assess whether FAKi can sensitize PDAC tumors to RT, we utilized a previously established RT-feasible KPC orthotopic model of PDAC^33^ to examine the impact of treatment with FAKi, RT, and their combination on tumor size. We inoculated KPC tumors orthotopically into mice on day 0. Tumor bearing mice started treatment on day 6 with IN10018 (designated FAKi below), which was administered once daily by oral gavage. Thirteen days after surgical implantation of KPC tumors into the pancreas, the mice started to receive hypofractionated RT (three daily doses at 8 Gy). Every 7 days, tumor volume measurements of all mice were taken using US (**Figure 1A**). We observed that over 45 days, there was no significant difference in tumor volume between untreated and FAKi-treated mice. However, RT-treated mice had a decrease in tumor size as compared to untreated mice and those treated with FAKi only. Additionally, mice treated with the combination of FAKi and RT, showed a significant decrease in tumor volume reduction as compared to each treatment alone or untreated mice (**Figure 1B**). This data suggests that FAKi alone has a negligible anti-tumor effect and that radiation alone as expected leads to a modest tumor reduction beyond untreated and FAKi-treated tumors. However, the addition of FAKi to RT leads to a significant reduction of tumor volume further suggesting the role of FAKi as a radiosensitizer.

### FAKi and RT combination treatment improves survival in murine model of PDAC

Next, we assessed whether radiosensitization, mediated by the addition of FAKi to RT, could translate into a survival advantage in KPC orthotopic mice. We again utilized our KPC orthotopic model, with a similar treatment strategy as above, and assessed the survival of each mice in each treatment group. Treatment with FAKi or RT alone did not result in any significant improvement in survival as compared to untreated mice. However, treatment with combination FAKi and RT did result in significant improvement in survival as compared to untreated and FAKi alone-treated mice. Although there was no statistically significant difference in survival of combination RT and FAKi compared to RT alone, we did appreciate a trend towards improved survival with the longest surviving mouse appreciated in the FAKi- and RT-treated group. These findings suggest that FAKi may indeed sensitize RT, leading to improvement in survival (**Figure 1C**).

### FAKi enhances CD8+ T cell infiltration in tumor treated by radiotherapy

**Figure 2:**
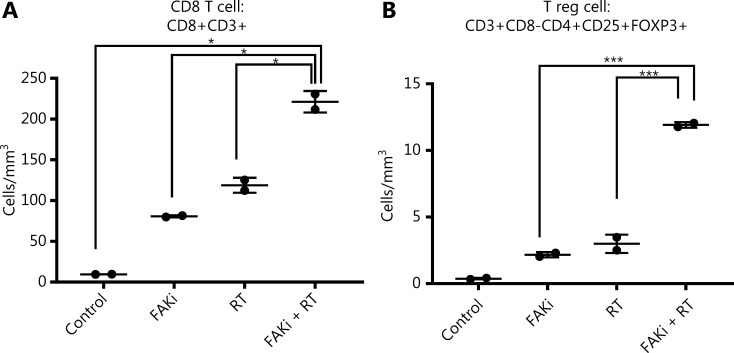
FAKi enhances CD8+ T cell infiltration in a tumor treated by radiotherapy. Following the inoculation of KPC tumor cells by orthotopic implantation, mice were treated with FAKi (50 mg/kg) by oral gavage once daily beginning on day 6 following surgery. On days 13–15, 8 Gy × 3 hypofractionated radiation was administered daily. Mice were sacrificed on day 20 and flow cytometry was performed on TIL isolated from processed dissected orthotopic tumors. Each experimental group consisted of three to four mice, pooled and analyzed individually in duplicates. T cell density was defined as cells/mm^3^ of each treatment group. Shown are (A) CD8 T cell (CD3+CD8+) density and (B) T reg cell density (CD3+CD8-CD4+CD25+FOXP3+). *P < 0.05, ***P < 0.001, by one-way ANOVA.

Previously Lee et al.^12^, proposed that RT driven tumor reduction is mediated by a CD8+ T cell immune response. Thus, we examined if FAKi could increase CD8+ infiltration into tumors treated by radiotherapy. Using our KPC orthotopic model, FAKi treatment was started on day 6 post-inoculation with KPC tumor cells. On day 13–15, mice received 8 Gy of RT. On day 20, all mice were sacrificed and orthotopic implanted tumors were harvested for FACS analysis of TILs. When assessing CD8+ T cell density, we appreciated a significant increase of infiltrating CD3+CD8+ T cell in mice with the addition of FAKi to RT as a combinatorial treatment compared to each treatment alone and in untreated mice (**Figure 2A**). This suggests that FAKi enhances CD8+ T cell infiltration in tumors treated by radiotherapy in PDAC murine models. We also observed that the addition of FAKi to RT, increased CD3+CD8-CD4+CD25+FOXP3+ T reg cell density (**Figure 2B**). The relative abundance of CD8+ T is much higher than that of T reg cells when FAKi in combination with RT.

### Targeting FAKi in combination with RT leads to myeloid cell modulation

**Figure 3:**
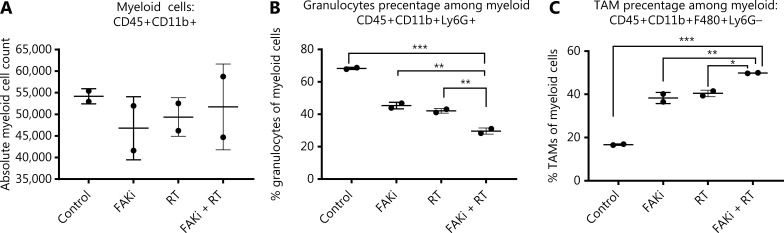
Targeting FAK in combination with RT leads to myeloid cell modulation. Mice were treated with FAKi (50 mg/kg) by oral gavage once daily beginning on day 6 following inoculation of KPC tumor cells by orthotopic implantation, then 8 Gy × 3 of hypofractionated radiation was given daily on days 13–15. Mice were sacrificed on day 20 and flow cytometry with CD11b+ cell isolation was performed on TIL separated from processed dissected orthotopic tumors. Each experimental group consisted of three to four mice, pooled and analyzed individually in duplicates. Shown are (A) absolute myeloid cell (CD45+CD11b+) count among all treatment groups, (B) granulocyte (CD45+CD11b+Ly6G+) percentage of myeloid cells, (C) TAM (CD45+CD11b+F480+Ly6G-) percentage of myeloid cells in each treatment group. ns, not significant, *P < 0.05, **P < 0.01, ***P < 0.001, by one-way ANOVA.

It has been proposed that myeloid cells, such as neutrophils, promote tumor resistance to RT and that myeloid phenotypes can modulate RT antitumor and immune response^36,37^. Thus, we next examined myeloid cell subsets to elucidate which phenotypes may modulate the CD8-mediated immune response associated with the addition of FAKi to RT-treated tissues, and the antitumor response appreciated with combination therapy. We found that there were no significant differences in absolute total CD45+CD11b+ myeloid cell counts with the addition of FAKi to RT (**Figure 3A**). However, when evaluating specific myeloid cell subsets, we found that the addition of FAKi to RT further decreased CD45+CD11b+Ly6G+ granulocyte percentage among the myeloid cell population as compared to FAKi and RT treatments alone (**Figure 3B**). The CD45+CD11b+F480+Ly6G- TAM percentage of myeloid cells was increased with the addition of FAKi to RT, as compared to each treatment alone (**Figure 3C**). These findings suggest the addition of FAKi to RT may modulate immunosuppressive granulocytes and possibly influence CD8+ T cell infiltration into the TME.

### FAKi and RT combination treatment improves antitumor response and survival in a murine model of PDAC in a T cell dependent fashion

**Figure 4:**
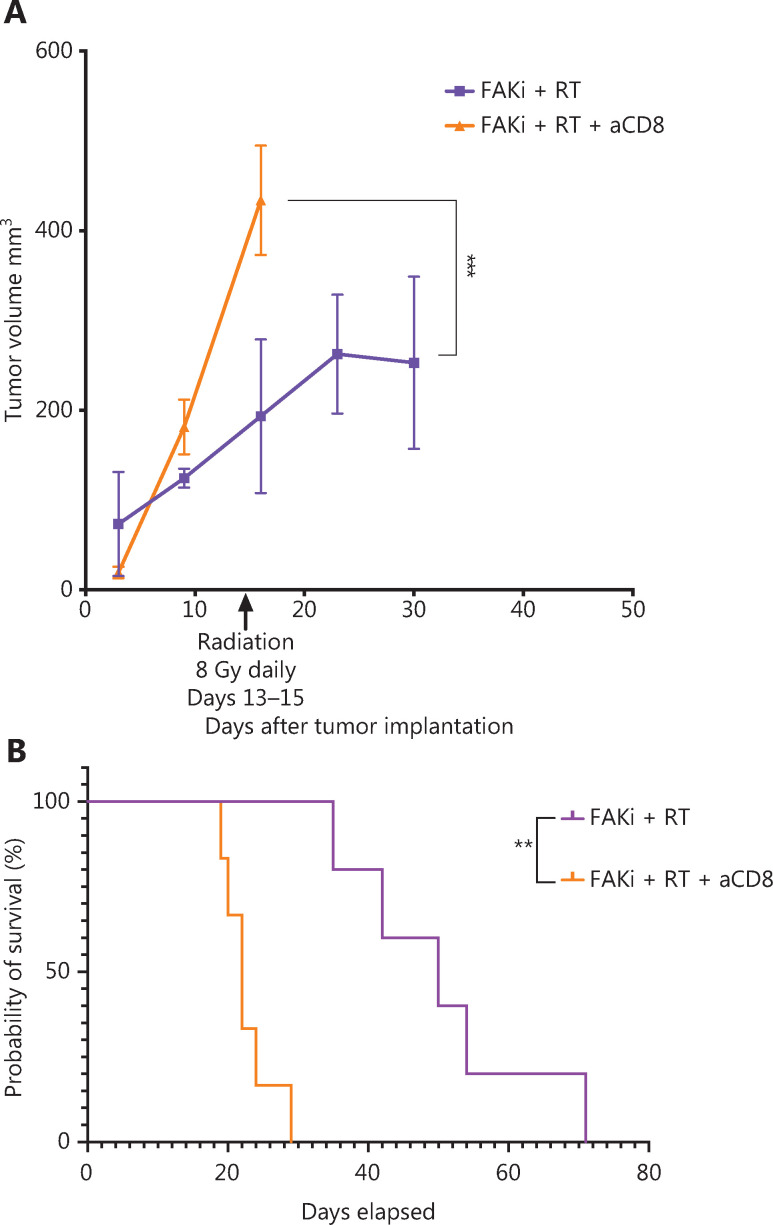
FAKi and RT combination treatment improves antitumor response and survival in a murine model of PDAC in a T cell dependent fashion. Following KPC tumor cell inoculation by orthotopic implantation, mice were depleted of CD8 cells starting on day 3 post-surgery, and then subsequently treated with FAKi (50 mg/kg) by oral gavage once daily beginning on day 6 following surgery. On days 13–15, 8 Gy × 3 of hypofractionated radiation was administered daily using surgical clips as radiation field markers. (A) US measurements in three dimensions were taken on days 3, 9, 16, 23, and 30 as noted on the x axis. Tumor volume represented as mm^3^ on the y axis was calculated using 3D US measurements within the transverse and sagittal planes. Each treatment group contained four to five mice. A two-way ANOVA with Šidák multiple comparisons test for each point was used. (B) Kaplan–Meier survival curves of mice implanted with PDAC cells treated with the combination FAKi and RT with CD8+ T-cell depletion (*n* = 6) and without CD8+ T-cell depletion (*n* = 5). ns, not significant, **P < 0.01, ***P < 0.001, by log-rank test.

The above findings suggest that enhanced antitumor activity by adding FAKi to RT is facilitated by CD8+ T cells. To test this hypothesis, we performed CD8+ T cell depletion starting on day 3 following orthotopic implantation in mice treated with FAKi and RT. We assessed both tumor volume and survival. We appreciated a significant increase in tumor size in FAKi and RT with CD8 depletion as compared to FAKi and RT without CD8 depletion (**Figure 4A**). We also noted that CD8 depletion led to significantly worse survival in FAKi- and RT-treated mice compared to those mice that received combination treatment without CD8 depletion (**Figure 4B**). In summary, these data suggest that FAKi serves as a radiotherapy sensitizer and enhances immune response in tissues treated with RT, leading to tumor reduction and improved survival in a T cell dependent manner.

## Discussion

Here, we present the first study of its kind evaluating the role of FAK inhibition as a radiotherapy sensitizer in PDAC. In this report we find that FAKi functions as a radiosensitizer, enhancing tumor reduction and improving survival in radiotherapy-treated murine PDAC. Additionally, we found that FAKi increases CD8+ T cell infiltration in PDAC tumors treated with radiotherapy and that the combination of FAKi and RT leads to modulation of myeloid phenotypes such as granulocytes. We also demonstrated that the antitumor response and survival advantage, appreciated with the combination of FAKi and RT in our murine model of PDAC, occurs in a T cell-dependent fashion, suggesting that FAK inhibition may enhance the antitumor response of RT in PDAC through an immune mechanism. FAKi as a radiosensitizer for the PDAC treatment warrants further clinical investigation.

It should be noted that, although FAKi, in combination with RT, slowed down the growth of primary PDAC tumors and lengthened the survival of the PDAC-bearing mice when comparing RT alone, none of mice survived at the end of the experiments. Although the exact cause of death was not investigated, especially in the experiments done in the current study, we suspected that the mice died from the metastatic diseases as we observed in the prior RT studies with the same model^33^. These results suggest that FAKi does not elicit adequate systemic therapeutic effects and highlights the importance of combining RT with systemic therapies in PDAC.

We found that granulocyte populations which can be immunosuppressive, were decreased by the addition of FAKi to RT. Whether this may account for the enhanced CD8+ T cell infiltration and antitumor response remain to be investigated. Previous studies suggested that FAK drives immunosuppressive cell modulation of CD8+ T cell infiltration by altering chemokine/cytokine networks^21,38^. It will be interesting to examine whether FAK inhibition may modulate the expression of the chemokine/cytokines in granulocytes in future studies. Moreover, our study also showed that the combination of FAKi and RT significantly enhanced the infiltration of TAMs and T regs in PDACs. Although it is possible that the antitumor M1 component of TAM, but not pro-tumoral M2 component, could be predominantly increased, and the increase of T regs could be a consequence of immune activation secondary to CD8+ T cell infiltration, it is more likely that FAK inhibition in combination with RT can induce compensative immunosuppressive mechanisms. Although it was not investigated in this study, our prior study, and the literature, suggested that RT (in the same mouse model of PDAC) would also induce PD-L1 expression and infiltration of T regs and TAMs^37,38^. In addition, we have previously shown that allogeneic PDAC vaccine, GVAX, can prime PDAC with effector T cell infiltration and upregulation of PD-1/PD-L1 for anti-PD-1/PD-L1 therapy^39^. Therefore, it will be interesting to study whether the combination of FAKi and RT, by enhancing CD8+ T cell infiltration, can prime PDAC for anti-PD-1/PD-L1 therapy. It will also be interesting to study whether FAK inhibition, by targeting granulocytic immunosuppressive cells, can be synergized with inhibition of other immunosuppressive pathways by combining immune checkpoint inhibitors, such as anti-PD-1/PD-L1 antibody and anti-CTLA4 antibody and with monocytes/macrophage targeting agents. To this end, our group has started to examine the combination of anti-PD-1 antibody and FAKi through a clinical trial of localized PDAC (NCT03727880). However, in future studies, RT should be considered as a testing component of this combination therapy for localized PDAC.

## Conclusions

In summary, our study reveals the novel therapeutic role of FAK inhibition as a potential radiosensitizer in PDAC. It also demonstrates the potential immunomodulatory effects of FAKi in PDAC treated with RT, as well as the ability of FAKi in combination with RT to prime the TME of PDAC with the infiltration of effector T cells. However, our investigation was limited by a small sample size due to the labor intensiveness of US examination and by a lack of additional syngeneic orthotopic models of PDAC available for the investigation of RT. Previously, it has been shown that the impact of FAKi on decreasing fibrosis in PDAC murine models and it is very likely that FAKi as radiosensitizer enhances CD8+ T Cell infiltration by modulating the fibroblast stroma^21^. This will be an important area for future investigation. Also, this study did not investigate the role of the tumor intrinsic response to FAKi and RT including the immunogenic cell death mechanism that may also mediate the enhanced CD8+ T cell infiltration in PDAC. Nevertheless, our findings have supported the rationale of exploring FAKi-mediated immune mechanisms for radiosensitivity in the preclinical models of PDAC, as well as other malignancies and including RT in human clinical trials evaluating FAKi in PDAC.
