# Supplementary material for: Inhibition of focal adhesion kinase enhances antitumor response of radiation therapy in pancreatic cancer through CD8+ T cells
Source: Cancer Biol Med. 2021 Feb 15;18(1):206–14. doi: 10.20892/j.issn.2095-3941.2020.0273 (PMC7877172; doi:10.20892/j.issn.2095-3941.2020.0273)
Supplement: Supplementary file 1 [file cbm-18-206-s001.pdf]

Supplementary material

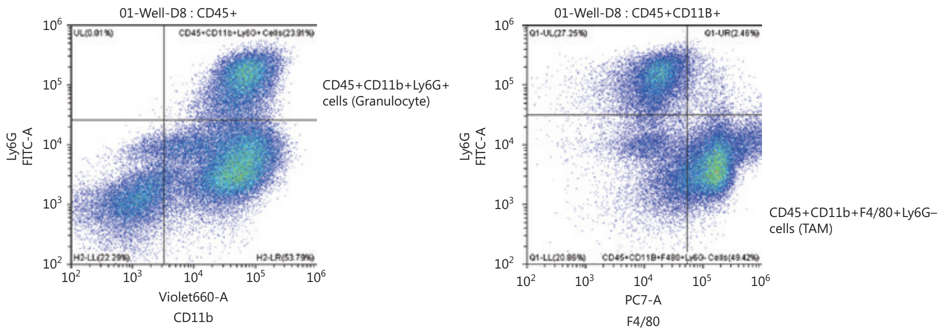

Figure S1 Gating Strategy for flow cytometry of myeloid phenotypes, Granulocytes and TAMs. Processed cells from each mouse were stained with Live Dead Aqua Dead Cell Kit Cells. The cells were then subsequently stained for the following anti-mouse fluorophores CD45-PERCP/Cy5.5, CD11b-BV650, Ly6G-FITC, and F4/80-PE/Cy7. Subsequently were then washed, resuspended in FACs buffer and assayed on a Cytoflex flow cytometer. Seen are the gating strategies for CD45+CD11b+Ly6G+ granulocytes (left) and CD45+CD11b+F480+Ly6G tumor associated macrophages (right).
